# Seroprevalence and molecular detection of *Toxoplasma gondii* and *Neospora caninum* in beef cattle and goats in Hunan province, China

**DOI:** 10.1186/s13071-024-06283-9

**Published:** 2024-04-26

**Authors:** Xi-Long Yi, Wen-He Yang, He-Liang Zheng, Mei-Ling Cao, Jun Xiong, Wen-Cheng Chen, Yu-Jing Zhou, Fen Li, Xing-Quan Zhu, Guo-Hua Liu

**Affiliations:** 1 Research Center for Parasites & Vectors, College of Veterinary Medicine, Hunan Agricultural University, Changsha, 410128 Hunan People’s Republic of China; 2 Chenzhou Center for Livestock Poultry and Aquatic Product Quality Inspection, Chenzhou, 423000 Hunan People’s Republic of China; 3 Laboratory of Parasitic Diseases, College of Veterinary Medicine, Shanxi Agricultural University, Taigu, 030801 Shanxi People’s Republic of China

**Keywords:** *Toxoplasma gondii*, *Neospora caninum*, Genotyping., China., Ruminants.

## Abstract

**Background:**

*Toxoplasma gondii* and *Neospora caninum* are closely related protozoan parasites that are considered important causes of abortion in livestock, causing huge economic losses. Hunan Province ranks 12th in the production of beef and mutton in China. However, limited data are available on the seroprevalence, risk factors and molecular characterization of *T. gondii* and *N. caninum* in beef cattle and goats in Hunan province, China.

**Methods:**

Sera of 985 beef cattle and 1147 goats were examined for the presence of specific antibodies against *T. gondii* using indirect hemagglutination test (IHAT) and anti-*N. caninum* IgG using competitive-inhibition enzyme-linked immunoassay assay (cELISA). Statistical analysis of possible risk factors was performed using PASW Statistics. Muscle samples of 160 beef cattle and 160 goats were examined for the presence of *T. gondii* DNA (B1 gene) and *N. caninum* DNA (Nc-5 gene) by nested PCR. The B1 gene-positive samples were genotyped at 10 genetic markers using the multilocus nested PCR-RFLP (Mn-PCR-RFLP).

**Results:**

Specific IgG against *T. gondii* were detected in 8.3% (82/985) and 13.3% (153/1147) and against *N. caninum* in 2.1% (21/985) and 2.0% (23/1147) of the beef cattle and goats, respectively. Based on statistical analysis, the presence of cats, semi-intensive management mode and gender were identified as significant risk factors for *T. gondii* infection in beef cattle. Age was a significant risk factor for *T. gondii* infection in goats (*P* < 0.05), and age > 3 years was a significant risk factor for *N. caninum* infection in beef cattle (*P* < 0.05). PCR positivity for *T. gondii* was observed in three beef samples (1.9%; 3/160) and seven chevon samples (4.4%; 7/160). Genotyping of PCR positive samples identified one to be ToxoDB#10. The *N. caninum* DNA was observed in one beef sample (0.6%; 1/160) but was negative in all chevon samples.

**Conclusions:**

To our knowledge, this is the first large-scale serological and molecular investigation of *T. gondii* and *N. caninum* and assessment of related risk factors in beef cattle and goats in Hunan Province, China. The findings provide baseline data for executing prevention and control of these two important parasites in beef cattle and goats in China.

**Graphical Abstract:**

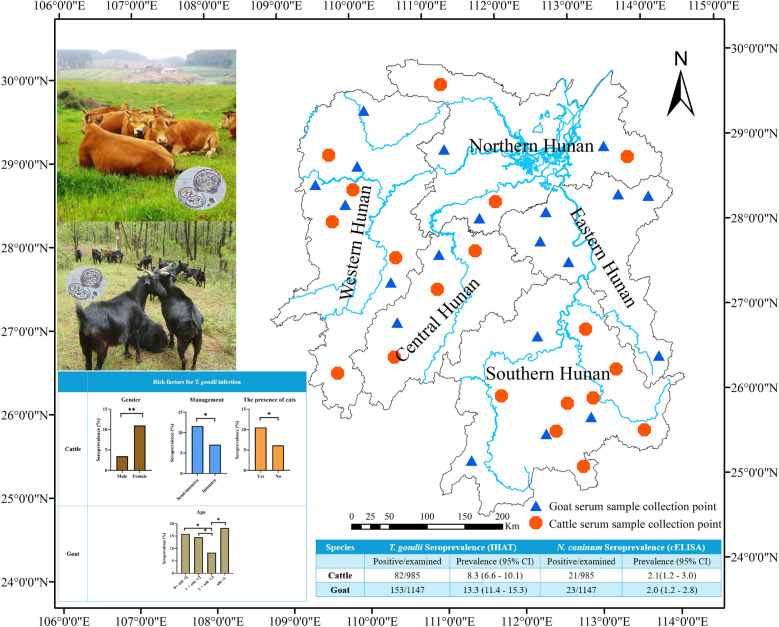

**Supplementary Information:**

The online version contains supplementary material available at 10.1186/s13071-024-06283-9.

## Background

Toxoplasmosis and neosporosis are two parasitic diseases with worldwide distribution, which are caused by *Toxoplasma gondii* and *Neospora caninum*, respectively [1]. Felids are the only definitive hosts of *T. gondii*, and all warm-blooded animals, including humans, can be infected as intermediate hosts [2]. Cattle and goats are two important intermediate hosts of *T. gondii* and *N. caninum*. *Toxoplasma gondii* and *N. caninum* are prevalent in ruminants worldwide, and they are associated with reproductive problems, particularly in cattle infected with *N. caninum* and in goats infected with *T. gondii*, causing significant economic losses [1, 3–5]. In China, the pooled seroprevalence of *T. gondii* in cattle and goats was 10.1% and 9.9%, respectively [6, 7]. The seroprevalence of *N. caninum* in cattle and goats varied in different regions of China, ranging from 9.4–20.9% to 3.9–7.2%, respectively [8, 9]. Infection in immunocompetent non-pregnant hosts is usually asymptomatic [10]. However, primary infection of humans and animals with *T. gondii* during pregnancy may result in abortion or congenital anomalies in the fetus [11]. In immunocompromised hosts, it may lead to severe generalized toxoplasmosis, which may even be fatal [12]. On the other hand, dogs are the most common definitive host of *N. caninum*, another important apicomplexan parasite in animals [3, 13, 14]. This parasite has been recognized as one of the most important causes of abortion in bovines worldwide [15]. In addition to dogs and cattle, natural infections have also been reported in goats, sheep, deer and horses, and these animals may also be potentially affected by *N. caninum* [16].

Although the average *T. gondii* prevalence in the Chinese population is relatively low, the total number of infected people is quite large because of the 1.4 billion total population of China [17]. An important route of infection in humans, next to contamination of food and water with oocysts, is consuming raw or undercooked meat (especially pork, chevon and beef) containing tissue cysts [18]. *Toxoplasma gondii* in cattle and goats is prevalent in some regions of China, which is a potential risk for transmitting the infection to humans. China is the major production and consumption market of beef and chevon worldwide, where production was about 7.18 million tons of beef and 5.25 million tons of mutton in 2022 (http://www.stats.gov.cn/tjsj/zxfb/202302/t20230227_1918980.html). Although *N. caninum* has not been proven to be a zoonotic pathogen, its impact on possible abortion and milk production in cattle is significant [13].

Hunan Province is located in the central-southern region of China, where its subtropical monsoon climate has endowed it with abundant vegetation. Additionally, the province boasts rich local breeds of cattle and goats, such as the Xiangxi Yellow Cattle, Xiangdong Black Goat and Matou Goat. These unique breeds have provided vast prospects for the livestock industry, further driving the expansion of the consumer market scale. According to statistics, beef and mutton production in Hunan Province has reached 356,500 tons, ranking 12th nationwide (https://m.voc.com.cn/xhn/news/202207/14095448.html). Considering the abundance of local breeds of cattle and goat in Hunan Province, as well as their potential in the breeding industry, investigations and molecular detection of *T. gondii* and *N. caninum* can play a crucial role in ensuring the quality and quantity of beef and mutton production in Hunan Province. However, the prevalence of these parasites in beef cattle and goats in Hunan Province has never been thoroughly investigated, with only one previous report [19]. Therefore, the objectives of the present study were to examine the seroprevalence and risk factors and to genetically characterize *T. gondii* and *N. caninum* in beef cattle and goats in Hunan Province, China.

## Methods

### Serum and muscle samples

**Fig. 1:**
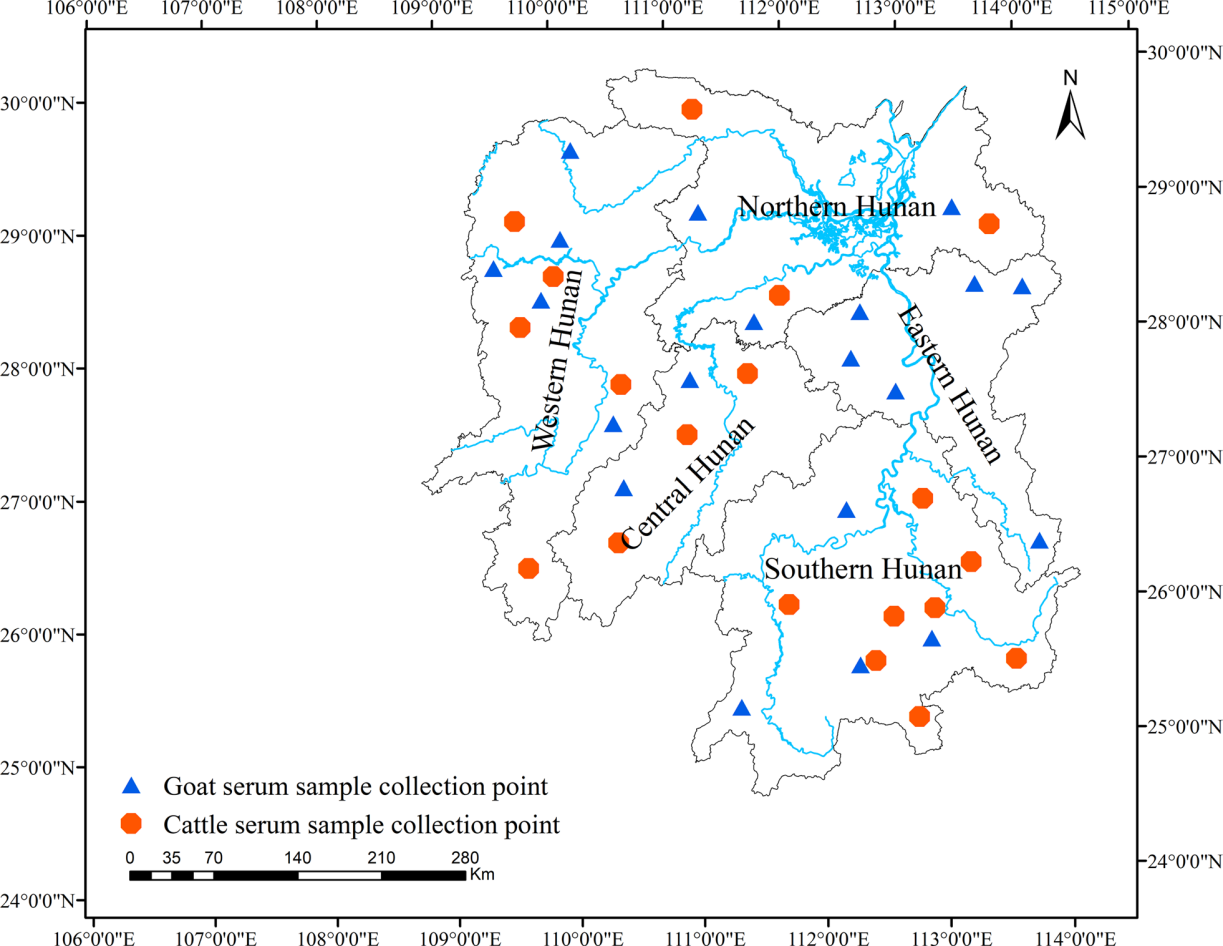
Sample collection sites for beef cattle and goat serum samples from different regions of Hunan Province are marked on the map using ArcGIS 10.8 software (ESRI Inc., Redlands, CA, USA). (http://www.esri.com/software/arcgis/arcgis-for-desktop). The red hexagon represents the collection point of cattle serum samples, and the blue triangle represents the collection point of goat serum samples

Based on geographical locations, Hunan Province is divided into five regions: central, western, eastern, southern and northern Hunan. In this study, a total of 985 beef cattle blood samples were collected from all regions except eastern Hunan (Fig. 1). These blood samples were collected from intensive farms as well as small holder farms. Meanwhile, 1147 goat blood samples were collected from intensive farms across the five regions of Hunan Province. The beef cattle and goats were randomly selected, with collection taking place between May 2018 and January 2019. Blood was drawn into glass tubes without anticoagulant from each animal on site. Samples were refrigerated and transferred to the laboratory to prepare sera and frozen at −20 °C until analysis. Information about the gender and age of the animals and the presence of free-roaming cats/dogs was recorded by local veterinarians.

A total of 320 intercostal muscle samples (160 beef and 160 chevon) were purchased from markets (farmers’ markets and supermarkets) in Hunan Province from September 2019 to November 2020. Each muscle tissue sample was cut into small pieces that weighed < 1 g and placed in a 2 ml centrifuge tube. The samples were then labeled and stored at −20 ℃.

### Serological examination

Specific antibodies against *T. gondii* in animals were detected using a commercial indirect hemagglutination test (IHAT) kit (Lanzhou Veterinary Research Institute, Chinese Academy of Agricultural Science, Lanzhou, China) according to the manufacturer’s instructions [20, 21]. The serum samples were deemed positive when an agglutination reaction was observed in wells diluted at ≥ 1:64. Antibodies (IgG) to *N. caninum* were examined by competitive-inhibition enzyme-linked immunoassay assay (cELISA) using ID Screen^®^ *N. caninum* Competition kit (IDVET, France) following the manufacturer’s recommendations [22]. Each ELISA plate included negative (nc) and positive controls (pc) provided by the manufacturer, and the samples' optical density (OD) was measured at 450 nm. The validity of the test was confirmed when both OD of negative control was > 0.700 and OD positive control/OD negative control < 0.3. For each sample, the competition percentage (S/N%) was determined using the formula: S/N % = (OD_sample_/OD negative control) × 100. Results were interpreted as follows: samples with S/N % ≤ 50% were considered positive, inconclusive if 50% < S/N % ≤ 60% and negative if S/N% > 60%.

### DNA extraction, PCR detection and genetic characterization

Total genomic DNA was extracted from 200 mg muscle samples from each sample purchased from markets using a commercia kit (Promega, USA). A semi-nested PCR targeting the B1 gene (131 bp) was used to examine the *T. gondii* DNA [23]. Although the sensitivity of the 529-bp repeat element is higher than that of the B1 gene, the B1 gene has been commonly used as genetic marker for the molecular detection of *T. gondii* DNA [24]. Positive (DNA extracted from GT1 strain) and negative (ultrapure water) controls were included in all PCR reactions. The B1 gene-positive samples were genotyped at 10 genetic markers [SAG1, SAG2 (5′ + 3′ SAG2, alter.SAG2), SAG3, BTUB, GRA6, c22–8, c29–2, L358, PK1 and Apico] using the multiplex multilocus nested polymerase chain reaction-restriction fragment length polymorphism technology (Mn-PCR-RFLP), and eight reference *T. gondii* strains (GT1, PTG, CTG, MAS, TgCgCa1, TgCatBr5, TgCatBr64 and TgRsCr1) were included as controls as described previously [25]. The PCR products were digested with specific restriction endonucleases, followed by electrophoretic separation of the resulting restriction fragments. Subsequently, typing data were analyzed using the ToxoDB database (http://toxodb.org/toxo/) and compared against reference strain profiles.

The Nc-5 gene was used to detect *N. caninum* infection as described previously [26]. Positive (*N. caninum* NC-1 strain) and negative (ultrapure water) controls were included in all PCR reactions. The PCR products were validated by electrophoresis using 1% agarose gels containing GoldView^™^ (Beijing, China). The positive PCR products were sequenced by Tsingke Biotech (Beijing, China). The sequences acquired were subjected to analysis and alignment using MEGA 7 software [27]. The aligned sequences were further assessed and compared through BLAST analysis.

### Statistical analysis

We categorized the samples by region, gender and age group (0–1, 1–2, 2–3 and > 3 years) for the analysis of potential infection risks. Additionally, as some beef cattle were semi-intensive, factors like presence of free-roaming cats/dogs and management modes (intensive or semi-intensive) were included for beef cattle samples. Variables associated with *T. gondii* and *N. caninum* infections among animals and different variables were analyzed using a chi-square test in PASW Statistics 18 (IBM Corporation, Somers, NY, USA), and 95% confidence intervals (CI) are also given. Probability (*P*) value < 0.05 was considered statistically significant.

## Results

Sera from 82 of 985 beef cattle (8.3%) and 153 of 1147 goats (13.3%) were positive for the presence of *T. gondii* antibodies. Twenty-one of 985 beef cattle (2.1%) and 23 of 1147 goats (2.0%) were positive for *N. caninum* antibodies. Co-infection of both *T. gondii* and *N. caninum* was detected in five of the 2132 (0.2%) animals, with three of 985 beef cattle (0.3%) and two of 1147 (0.2%) goats being infected (Table 1).

**Table 1:** Seroprevalence of *Toxoplasma gondii* and *Neospora caninum* infection in cattle and goats in Hunan province, China

Species	*T. gondii* seroprevalence (IHAT)		*N. caninum* seroprevalence (cELISA)		*T. gondii* + *N. caninum* seroprevalence	
	Positive/examined	Prevalence (95% CI)	Positive/examined	Prevalence (95% CI)	Positive/examined	Prevalence (95% CI)
Cattle	82/985	8.3 (6.6—10.1)	21/985	2.1 (1.2—3.0)	3/985	0.3 (0–0.6)
Goat	153/1147	13.3 (11.4—15.3)	23/1147	2.0 (1.2—2.8)	2/1147	0.2 (0–0.4)

*IHAT* indirect hemagglutination test, *cELISA* competitive-inhibition enzyme-linked immunoassay assay

The analysis results of possible risk factors of *T. gondii* infection in beef cattle and goats in Hunan Province are shown in Table 2 and Table 3. The presence of cats, semi-intensive management mode and gender were considered important risk factors for *T. gondii* infection in beef cattle (*P* < 0.05). The overall risk of cows being infected with *T. gondii* is nearly four times that of bulls (OR = 3.5, *P* < 0.001). The risk of *T. gondii* infection in beef cattle raised with cats in the environment is nearly twice that of beef cattle without cats (OR = 1.8, *P* < 0.05). The risk factors of semi-intensively raised beef cattle being infected with *T. gondii* were nearly two times those of intensively raised beef cattle (OR = 1.7, *P* < 0.05). In addition, for beef cattle in different regions of Hunan Province, the risk of beef cattle being infected with *T. gondii* in southern Hunan is nearly four times that in central Hunan (OR = 3.8, *P* < 0.05), and the risk of beef cattle being infected with *T. gondii* in northern Hunan is nearly three times of that in central Hunan (OR = 2.8, *P* < 0.05). Other factors including age were not statistically significant (*P* > 0.05).

**Table 2:** Analysis of risk factors for *Toxoplasma gondii* infection in cattle in Hunan Province, China

Factor	Category	No. tested	Prevalence (%)	OR (95%CI)	*P*-value
Region	Western hunan	143	8.39	2.8 (0.9—9.0)	> 0.05
	Central hunan	128	3.13	Reference	
	Southern hunan	**287**	**10.80**	**3.8 (1.3—10.9)**	** < 0.05**
	Northern hunan	**427**	**8.20**	**2.8 (1.0—7.9)**	** < 0.05**
Gender	Male	348	3.45	Reference	
	Female	**637**	**10.99**	**3.5 (1.8—6.5)**	** < 0.05**
Age	0 < year < 1	72	11.11	2.0 (0.7—6.0)	> 0.05
	1 < years < 2	486	8.02	1.4 (0.6—3.4)	> 0.05
	2 < years < 3	325	8.92	1.6 (0.6—3.9)	> 0.05
	Years > 3	102	5.88	Reference	
Presence of free-roaming cat	Yes	**495**	**10.51**	**1.8 (1.1—2.9)**	** < 0.05**
	No	490	6.12	Reference	
Management mode	Intensive	709	7.05	Reference	
	Semi-intensive	**276**	**11.59**	**1.7 (1.1—2.8)**	** < 0.05**

Bold values indicate statistical significance at the *P* < 0.05 level

**Table 3:** Analysis of risk factors for *Toxoplasma gondii* infection in goats in Hunan Province, China

Factor	Category	No. tested	Prevalence (%)	OR (95%CI)	*P*-value
Region	Central Hunan	191	8.38	Reference	Reference
	Eastern Hunan	310	13.87	1.76 (0.96—3.23)	> 0.05
	Southern Hunan	206	12.14	1.51 (0.78—2.93)	> 0.05
	Western Hunan	**312**	**17.63**	**2.34 (1.30—4.22)**	** < 0.05**
	Northern Hunan	128	10.94	1.34 (0.63—2.86)	> 0.05
Gender	Male	152	9.87	Reference	Reference
	Female	995	13.87	1.05 (0.84—2.58)	> 0.05
Age	0 < year ≤ 1	**228**	**15.79**	**2.09 (1.21—3.59)**	** < 0.05**
	1 < years ≤ 2	**539**	**14.47**	**1.88 (1.17—3.02)**	** < 0.05**
	2 < years ≤ 3	303	8.25	Reference	Reference
	Years > 3	**77**	**18.18**	**2.47 (1.22—5.02)**	** < 0.05**

Bold values indicate statistical significance at the *P* < 0.05 level

For goats, we found that age was statistically significant among all possible risk factors for *T. gondii* infection. The seroprevalences of goats aged 0–1 (OR = 2.09, *P* < 0.05), 1–2 (OR = 1.88, *P* < 0.05) and > 3 years old (OR = 2.47, *P* < 0.05) are nearly twice those of goats aged 2 to 3 years old. In addition, for goats in different regions of Hunan Province, the risk of goats being infected with *T. gondii* in western Hunan is nearly two times that in central Hunan (OR = 2.34, *P* < 0.05).

The results of analysis of risk factors for *N. caninum* seroprevalence in beef cattle and goats in Hunan Province are shown in Additional file 1: Tables S1, S2. It was found that age > 3 years is a statistically significant risk factor for *N. caninum* seroprevalence in beef cattle (*P* < 0.05). Additionally, seroprevalence may vary between regions in goats. Goats in central and western Hunan have much higher risks [nearly 10 times (OR = 10.02, *P* < 0.05) and 12 times (OR = 12.36, *P* < 0.05), respectively] of being infected with *N. caninum* compared to goats in eastern Hunan.

PCR positivity for *T. gondii* was observed in three beef samples (1.9%; 3/160) and seven chevon samples (4.4%; 7/160) targeting the B1 gene. Only one of the three positive DNA samples provided data at all genetic loci and was identified as ToxoDB#10 (Table 4). However, the genotypes of seven chevon samples remained uncertain because of the ambiguity associated with two genetic markers. The *N. caninum* DNA was amplified in one beef sample (0.6%; 1/160) but was negative in all chevon samples. The Nc-5 sequence obtained in this study (accession no. MW590805) had 98.33% identity to a previously published corresponding *N. caninum* sequence (accession no. KP715562).

**Table 4:** Genotyping of *Toxoplasma gondii* isolates in cattle in Hunan Province, China

Isolate ID	SAGI	5′ + 3′ SAG2	Alternative SAG2	SAG3	BTUB	GRA6	c22–8	c29–2	L358	PK1	Apico	Genotype
GT1^a^	I	I	I	I	I	I	I	I	I	I	I	ToxoDB#10
PTG^a^	II/III	II	II	II	II	II	II	II	II	II	II	ToxoDB#1
CTG^a^	II/III	III	III	III	III	III	III	III	III	III	III	ToxoDB#2
MAS	u-1^b^	I	II	III	III	III	u-1^b^	I	I	I	I	ToxoDB#17
TgCgCa1	I	II	II	III	II	II	II	u-1^b^	I	u-2^b^	I	ToxoDB#66
TgCatBr5	I	III	III	III	III	III	I	I	I	u-1^b^	I	ToxoDB#19
TgCatBr64	I	I	u-1^b^	III	III	III	u-1^b^	I	III	III	I	ToxoDB#111
TgRsCr1	u-1^b^	I	II	III	I	III	u-2^b^	I	I	I	I	ToxoDB#52
HNC39 (Cattle)	**I**	**I**	**I**	**I**	**I**	**I**	**I**	**I**	**I**	**I**	**I**	**ToxoDB#10**

^a^Clonal genotypes I (GT1), II (PTG) and III (CTG).

^b^u-1 and u-2 represent unique RFLP genotypes, respectively

Bold text represents the sample we studied

## Discussion

This study revealed the widespread presence of *T. gondii* and *N. caninum* infections in common ruminants in Hunan Province. The present and previous results showed the presence of *T. gondii* infection in beef and chevon in China, posing a potential threat to human health [28, 29]. In certain regions of China, some people like to eat raw beef because of the mistaken belief that raw beef is highly nutritious [30]. Therefore, the risk of *T. gondii* infection in humans is greatly increased by eating raw and undercooked infected meat (beef and chevon).

In this study, serological assays showed that co-infection of beef cattle (0.3%) and goats (0.2%) with both parasites was rare, which is consistent with the results of many previous studies [21, 31, 32]. However, seroprevalence of *T. gondii* and *N. caninum* in cattle and goat varied greatly between countries and between different regions within the same country. The seroprevalence rate of *T. gondii* infection in beef cattle reported in this study (8.3%) is roughly similar to that in central Ethiopia (6.6%) and Indonesia (9%) but lower than that in Algeria (28.7%) and Libya (27.4%) [33–36]. We also compared the seroprevalence of *T. gondii* in cattle between Hunan Province and other provinces in China. The prevalence is higher than that previously found in Liaoning (6.0%) and Guangdong (5.7%) in China but is significantly lower than that reported in Guizhou (26.9%), Chongqing (27.2%), Qinghai (35.5%) and Xinjiang (46.4%) [37].

In goats, the seroprevalence of *T. gondii* infection observed here (13.3%) was similar to that reported in central Ethiopia (11.6%) but lower than in Algeria (33.6%), Pakistan (30%) and Egypt (38.2%) [33, 34, 38, 39]. The seroprevalence of *T. gondii* infection in goats in Hunan Province was higher than that found in Inner Mongolia (7.9%), Jiangxi (10.3%) and Heilongjiang (3.8%) but was significantly lower than that in Chongqing (42.5%), Yunnan (34.9%) and Shanxi (29.8%) [40]. These variations in seroprevalence may be related to the diagnostic techniques, climate, management mode of the farm and geographical conditions [41].

Our study showed that the *N. caninum* seroprevalence in beef cattle (2.1%) was similar to that in Malaysia (2.7%) but significantly lower than that in Argentina and Serbia (7.2%) [42–45]. In addition, it is also significantly lower than the average seroprevalence of *N. caninum* in cattle in China (13.69%) [46]. The lower seroprevalence of *N. caninum* in cattle in this study may be because the serum samples in this study were all from beef cattle. Some studies have shown that beef cattle are less susceptible to *N. caninum* infection than dairy cattle, so their seroprevalence rates tend to be lower [44, 47, 48]. The seroprevalence rate in goats (2%) is similar to that in beef cattle in this study and is also significantly lower than that of France (33%), Spain (6%) and Malaysia (3.9%) and significantly lower than that in Qinghai Province (7.2%), China [31, 32, 45, 49]. Furthermore, our study showed that the difference in *N. caninum* prevalence between beef cattle and goats was not significant, similar to a previous study [31]. The low *N. caninum* seroprevalence rates in beef cattle and goat in Hunan Province may be related to climate, geographical environment, the good management mode of the farm or different study designs and diagnostic methods [31, 43, 44, 50].

In addition to the study of seroprevalence, we also analyzed risk factors that can help formulate relevant prevention and control strategies. Some of the risk factors for *T. gondii* infection in beef cattle and goats demonstrated statistical significance. Among the beef cattle in this study, higher *T. gondii* seroprevalence was observed in cows, in semi-intensive management and on farms with cats. Our study demonstrated that cows exhibited higher seroprevalence than bulls, which could potentially be attributed to hormonal differences related to gender that influence immune responses against *T. gondii* [33, 41, 51, 52]. Semi-intensive raising and the presence of cats around the farm have been reported in many previous studies as risk factors for *T. gondii* infection [45, 53–55], and these factors increase the probability and frequency/chances of exposure of cattle to oocysts that are widely distributed in the environment. In addition, the seroprevalence of *T. gondii* in beef cattle in central Hunan was about two times lower than that in other areas of Hunan Province. There is a statistically significant difference in the seroprevalence of *T. gondii* in goats between western and other regions Hunan. Also, we observed a statistically significant difference in the seroprevalence of *N. caninum* between central Hunan and western Hunan compared to other regions. This variation may be due to differences in animal welfare, climate, investigation methods, animal husbandry practices and geography [41]. Among the goats in this study, significant differences in *T. gondii* seroprevalence were observed among goats of different ages. Taking the age group of 2 to 3 years as a reference, the *T. gondii* seroprevalences in goats aged 0–1, 1–2 and > 3 years old are nearly twice that in goats aged 2 to 3 years old, showing a trend of lowering first and then getting higher. This trend is different from most studies, which indicate that seroprevalence increases with age [33, 38, 41, 56]. The trend may be due to the following reasons. For newborn lambs from younger ewes, vertical transmission may be one of the reasons for the relatively high *T. gondii* antibody levels at this age. On the one hand, *T. gondii* is transmitted from the ewe to the fetus via the placenta, and lambs are born with circulating antibodies. On the other hand, tachyzoites of *T. gondii* may be present in breast milk, which may cause infection after suckling. Of course, among the possible reasons for the high antibody levels, it cannot be ruled out that lambs received maternal antibodies against *T. gondii* from colostrum [57, 58]. For older goats, longer exposure time to oocysts in the environment may be related to their higher antibody levels, and the same phenomenon was observed in our study of beef cattle > 3 years old infected with *N. caninum* [33, 59].

Previous studies have identified ToxoDB#225 and ToxoDB#10 in cattle in Henan and Jilin Provinces [37] and ToxoDB#9 and ToxoDB#10 in goats in Yunnan Province in China [60]. Considering previous molecular reports in Hunan Province, ToxoDB#10 was also identified from wild birds [61]. However, in this study, only one genotype (ToxoDB#10) was identified in one beef sample. The genotyping results from chevon samples were only suggestive because of the uncertainty of two genetic markers. ToxoDB#9 (Chinese 1) and ToxoDB#10 (Type I) are considered the predominant genotypes of *T. gondii* in China, and our detection of ToxoDB#10 in beef provides new evidence supporting this view [62, 63]. In addition, our study only detected one positive sample of *N. caninum* collected from a farmer's market in Changsha City. This is the first report of amplification of *N. caninum* Nc-5 gene in beef cattle muscle tissue from Hunan Province. The number and sources of samples need to be further expanded in the future to obtain more information on the genotypes of *T. gondii* and *N. caninum* in infected beef cattle and goats.

## Conclusions

This study represents the first extensive serological and molecular examination of *T. gondii* and *N. caninum* infection and associated risk factors in beef cattle and goat populations in Hunan Province, China. Our results may provide baseline information for the development of control measures against these parasitic infections in beef cattle and goats in this province and elsewhere. Future studies are necessary to elucidate the potential effect of both *T. gondii* and *N. caninum* on reproduction of ruminants. It is recommended to implement good management measures on ruminant farms to decrease the *T. gondii* infection in cattle and goats.

### Supplementary Information

**Additional file 1: Table S1.** Analysis of risk factors for *Neospora caninum* infection in beef cattle in Hunan Province, China. **Table S2.** Analysis of risk factors for *Neospora caninum* infection in goats in Hunan Province, China.

## Data Availability

The datasets supporting the findings of this article are included within the paper and its supplementary materials. The Nc-5 sequence of *Neospora caninum* obtained in this study is available at GenBank (accession no. MW590805).

## Abbreviations

IHAT: Indirect hemagglutination test
cELISA: Competitive-inhibition enzyme-linked immunoassay assay
OD: Optical density
S/N%: Competition percentage
PCR: Polymerase chain reaction
Mn-PCR–RFLP: Multilocus nested polymerase chain reaction-restriction fragment length polymorphism technology
OR: Odds ratio
CI: Confidence interval
