# Supplementary material for: Seroprevalence and molecular detection of Toxoplasma gondii and Neospora caninum in beef cattle and goats in Hunan province, China
Source: Parasit Vectors. 2024 Apr 26;17:195. doi: 10.1186/s13071-024-06283-9 (PMC11055231; doi:10.1186/s13071-024-06283-9)
Supplement: Supplementary file 1 — Additional file 1: Table S1. Analysis of risk factors for Neospora caninum infection in beef cattle in Hunan Province, China. Table S2. Analysis of risk factors for Neospora caninum infection in goats in Hunan Province, China. [file 13071_2024_6283_MOESM1_ESM.docx]

**Additional file 1: Table S1.** Analysis of risk factors for *Neospora caninum* infection in beef cattle in Hunan Province, China

| **Factor** | **Category** | **No. tested** | **Prevalence (%)** | **OR (95%CI）** | ***P*-value** |
| --- | --- | --- | --- | --- | --- |
| Region | Western Hunan | 143 | 2.80 | 2.4 (0.6 - 9.2) | > 0.05 |
|  | Central Hunan | 128 | 1.56 | 1.3 (0.3 - 7.0) | > 0.05 |
|  | Southern Hunan | 287 | 3.48 | 3.0 (1.0 - 9.0) | > 0.05 |
|  | Northern Hunan | 427 | 1.17 | Reference |  |
| Gender | Male | 348 | 1.72 | Reference |  |
|  | Female | 637 | 2.35 | 1.4 (0.5 - 3.6) | > 0.05 |
| Age | 0 < year ≤ 1 | 72 | 4.17 | 3.5 (0.8 - 15.9) | > 0.05 |
|  | 1 < years ≤ 2 | 486 | 1.65 | 1.3 (0.4 - 4.5) | > 0.05 |
|  | 2 < years ≤ 3 | 325 | 1.23 | Reference |  |
|  | **Years > 3** | **102** | **5.88** | **5.0 (1.4 - 18.1)** | **< 0.05** |
| The presence of free-roaming dogs | Yes | 568 | 2.29 | 1.2 (0.5 - 2.9) | > 0.05 |
|  | No | 417 | 1.92 | Reference |  |
| Management mode | Intensive | 709 | 1.70 | Reference |  |
|  | Semi-intensive | 276 | 3.26 | 2.0 (0.8 - 4.7) | > 0.05 |

| **Factor** | **Category** | **No. tested** | **Prevalence (%)** | **OR (95%CI）** | ***P*-value** |
| --- | --- | --- | --- | --- | --- |
| Region | **Central Hunan** | **191** | **3.14** | **10.02 (1.20 - 83.90)** | **< 0.05** |
|  | Eastern Hunan | 310 | 0.32 | Reference | Reference |
|  | Southern Hunan | 206 | 0.49 | 1.51 (0.09 - 24.23) | > 0.05 |
|  | **Western Hunan** | **312** | **3.85** | **12.36 (1.60 - 95.64)** | **< 0.05** |
|  | Northern Hunan | 128 | 2.34 | 7.42 (0.76 - 71.98) | > 0.05 |
| Gender | Male | 152 | 4.61 | 2.95 (1.20 - 7.30) | > 0.05 |
|  | Female | 995 | 1.61 | Reference | Reference |
| Age | 0 < year ≤ 1 | 228 | 1.75 | 1.19 (0.35 - 3.98) | > 0.05 |
|  | 1 < years ≤ 2 | 539 | 1.48 | Reference | Reference |
|  | 2 < years ≤ 3 | 303 | 2.31 | 1.57 (0.56 - 4.37) | > 0.05 |
|  | Years > 3 | 77 | 5.19 | 3.64 (1.07 - 12.38) | > 0.05 |

**Additional file 1: Table S2.** Analysis of risk factors for *Neospora caninum* infection in goats in Hunan Province, China
